# pMAA-Red: a new pPZP-derived vector for fast visual screening of transgenic Arabidopsis plants at the seed stage

**DOI:** 10.1186/1472-6750-12-37

**Published:** 2012-07-02

**Authors:** Muhammad Amjad Ali, Kausar Hussain Shah, Holger Bohlmann

**Affiliations:** 1 Division of Plant Protection, Department of Crop Sciences, University of Natural Resources and Life Sciences, Vienna, Austria; 2 Division of Plant Protection, Department of Crop Sciences, University of Natural Resources and Life Sciences, UFT Tulln, Konrad Lorenz Strasse 24, 3430, Tulln, Austria

**Keywords:** Transient expression, pPZP family vectors, Marker gene, Agroinfiltration, DsRed, Agrobacterium, Arabidopsis transformation

## Abstract

**Background:**

The production of transgenic plants, either for the overproduction of the protein of interest, for promoter: reporter lines, or for the downregulation of genes is an important prerequisite in modern plant research but is also very time-consuming.

**Results:**

We have produced additions to the pPZP family of vectors. Vector pPZP500 (derived from pPZP200) is devoid of NotI sites and vector pPZP600 (derived from pPZP500) contains a bacterial kanamycin resistance gene. Vector pMAA-Red contains a *Pdf2.1*: DsRed marker and a CaMV:: GUS cassette within the T-DNA and is useful for the production of promoter: GUS lines and overexpression lines. The *Pdf2.1* promoter is expressed in seeds and syncytia induced by the beet cyst nematode *Heterodera schachti* in Arabidopsis roots*.* Transgenic seeds show red fluorescence which can be used for selection and the fluorescence level is indicative of the expression level of the transgene. The advantage is that plants can be grown on soil and that expression of the marker can be directly screened at the seed stage which saves time and resources. Due to the expression of the *Pdf2.1*: DsRed marker in syncytia, the vector is especially useful for the expression of a gene of interest in syncytia.

**Conclusions:**

The vector pMAA-Red allows for fast and easy production of transgenic Arabidopsis plants with a strong expression level of the gene of interest.

## Background

Modern plant research relies heavily on the use of transient and stable transformation with the help of *Agrobacterium tumefaciens*[1,2]. This bacterium is a natural genetic engineer and transfers a part of its large Ti plasmid into host plants to induce cell division and the synthesis of opines. For use in genetic engineering, the Ti plasmid has been divided into a helper plasmid which is devoid of the T-DNA and remains within the Agrobacteria and a binary vector carrying the T-DNA which can be manipulated in *E. coli*. The first widely used binary vector was pBIN19 [3]. Many derivatives have been described and some are still in use today although other binary vectors which are smaller, have a higher copy number, and different selectable markers for use in bacteria (*E. coli* and Agrobacteria) and in plants have been introduced (for review see [4,5]). One popular series are the pPZP vectors [6] which were also the basis for the pCAMBIA vectors [7]. We have recently published an improved pPZP vector (pPZP3425) which was equipped with a kanamycin resistance gene for selection in Agrobacterium [8] . The strong 35 S CaMV promoter driving the plant resistance gene for kanamycin resistance was replaced by the weaker *nos* promoter because it had been shown that the 35 S promoter driving the plant resistance marker in the original pPZP vectors can lead to ectopic expression of the transgene [9,10]. Furthermore, pPZP3425 contains an expression cassette which consists of an intron-containing *GUS* gene driven by a strong constitutive promoter (35 S promoter with doubled enhancer plus omega element as translational enhancer). This vector has successfully been used in our laboratory.

Plant selectable markers for the pPZP vectors include kanamycin and gentamycin. Both markers work well for a variety of plant species. Kanamycin is perhaps the most widely used selectable marker for plant transformation. Kanamycin and gentamycin as well as other antibiotic markers have the disadvantage that they are usually used under sterile conditions. In case of Arabidopsis this means that to isolate transgenic plants the seeds have to be sterilized and grown on a sterile agar medium containing the antibiotics. Recently it has been shown that the selection of transgenic plant lines containing a kanamycin marker gene can be done by culturing the seedlings on rockwool saturated with MS medium without sugar but containing the selective agent [11]. Since the medium does not contain sugar, sterile conditions are not necessary, saving costs and labour. However, extreme care has to be taken that the seedlings do not run dry. Other markers that also circumvent the need to work under sterile conditions use resistance against herbicides, especially phosphinotricin (BASTA). The herbicide can be sprayed onto plants growing in soil to select for those containing the *Bar* gene which mediates resistance against phosphinotricin [12]. Fluorescent proteins have also been reported as markers for plant transformation including Arabidopsis [13-17]. For Arabidopsis transformation, DsRed, GFP, and GFP variants have been used as markers driven by seed-specific promoters derived from other plant species [18,19].

During cloning of a vector for transient expression we realized that the pPZP vectors contain 3 NotI sites in their backbone such that this eight-cutter could not be used in the polylinker. Starting with pPZP200, we have therefore removed all NotI sites from the vector backbone as well as other unnecessary parts to produce pPZP500. By replacing the spectinomycin resistance gene with the *nptII* gene we also produced the vector pPZP600.

The vector pPZP500 does not contain a plant selectable marker as this is not needed for transient expression. However, since pPZP500 was much smaller than the original pPZP vectors, it could be the basis of a new binary vector (pMAA-Red) for stable transformation of Arabidopsis. For that we included a DsRed gene driven by the *Pdf2.1* promoter and the GUS cassette from pPZP3425. The *Pdf2.1* promoter was chosen because it is strongly expressed in seeds and in syncytia, feeding sites induced by the beet cyst nematode *H. schachtii* in Arabidopsis roots [20,21]. In addition, we replaced the spectinomycin resistance gene used for selection in Agrobacteria by a kanamycin resistance gene.

## Results

### Construction of pPZP500 and pPZP600

**Figure 1:**
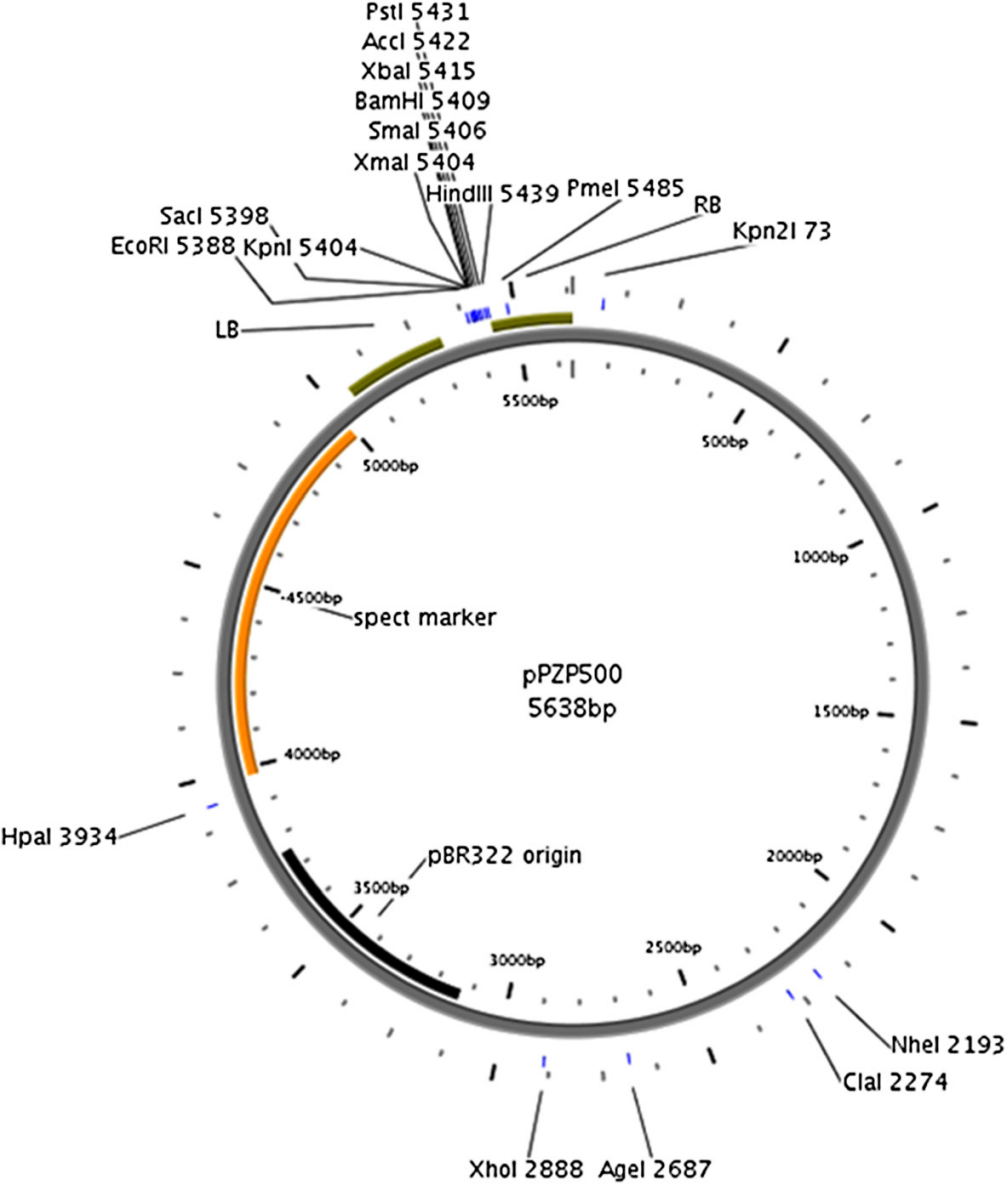
**pPZP500. **Circular map with main restriction sites, polylinker, and pBR322 origin. LB, left border; RB, right border; spect marker, spectinomycin marker.

**Figure 2:**
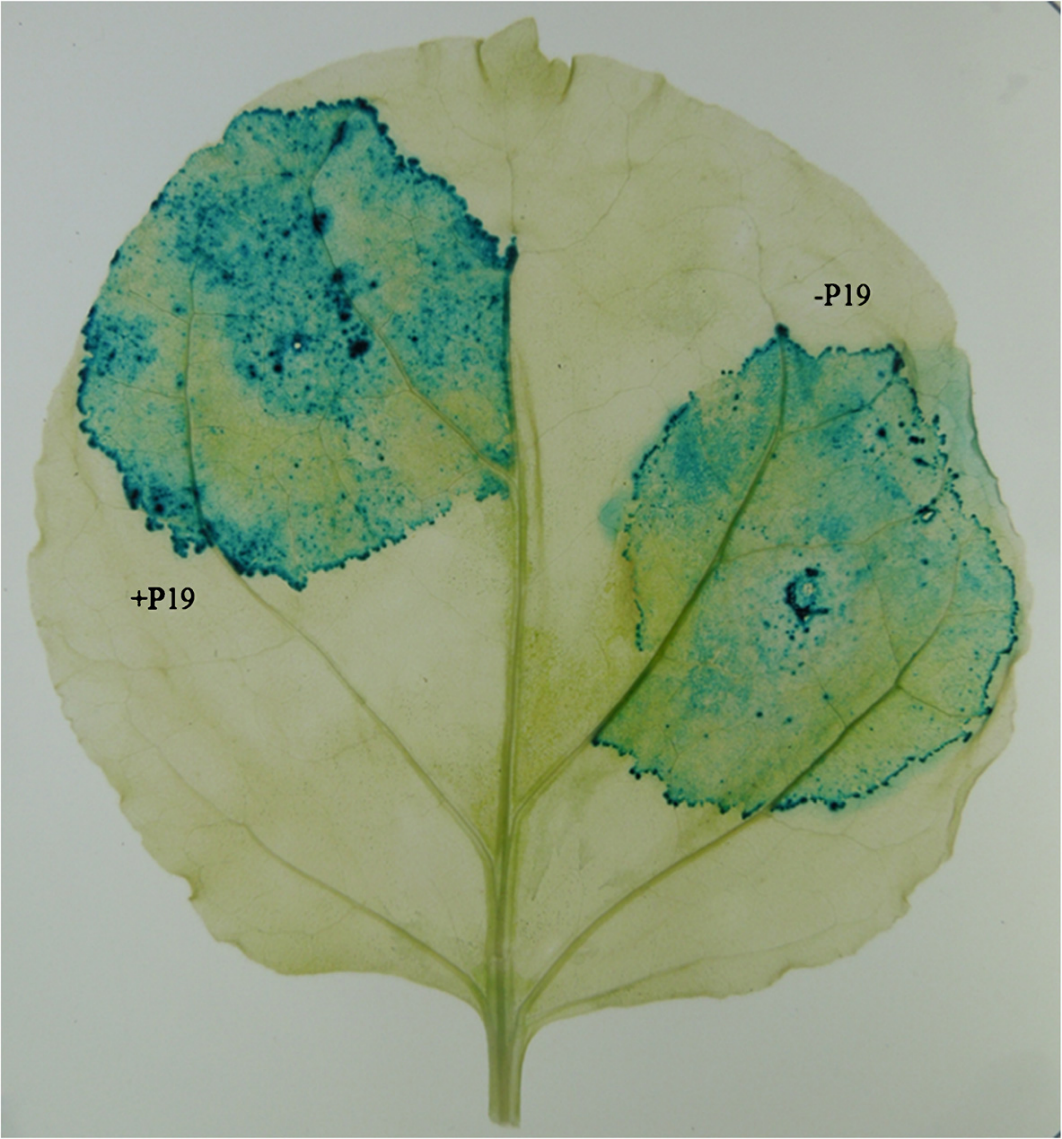
**GUS expression from pPZP5025. ***Nicotiana benthamiana *leaves were infiltrated with Agrobacteria harbouring pPZP5025 and stained for GUS expression 5 days later.

The binary vector pPZP200 is a high copy number, stable, and fully sequenced plasmid vector harbouring the pVSI derived backbone [6]. However, presence of three NotI sites in pPZP200 precluded the use of NotI for cloning. Therefore, these NotI sites have been eliminated by a series of PCR amplifications (Additional file 1: Figure S1) to produce pPZP500 (Figure 1) as described in the Methods section. We confirmed that the vector was still fully functional by introduction of the GUS cassette from pPZP3425 [8] and transient expression of the resulted plasmid pPZP5025 in *Nicotiana benthamiana* (Figure 2). 

Vector pPZP500 contains the streptomycin/spectinomycin resistance gene (*aadA,* encoding aminoglycoside-3"-adenyltransferase) which can be used in *E. coli* and in Agrobacteria. However, in our hands spectinomycin selection was not as tight as kanamycin selection. We, therefore, replaced the spectinomycin resistance gene with the *nptII* gene which yielded the vector pPZP600 (Additional file 2: Figure S2) as described in Methods.

### Construction of pMAA-Red

**Figure 3:**
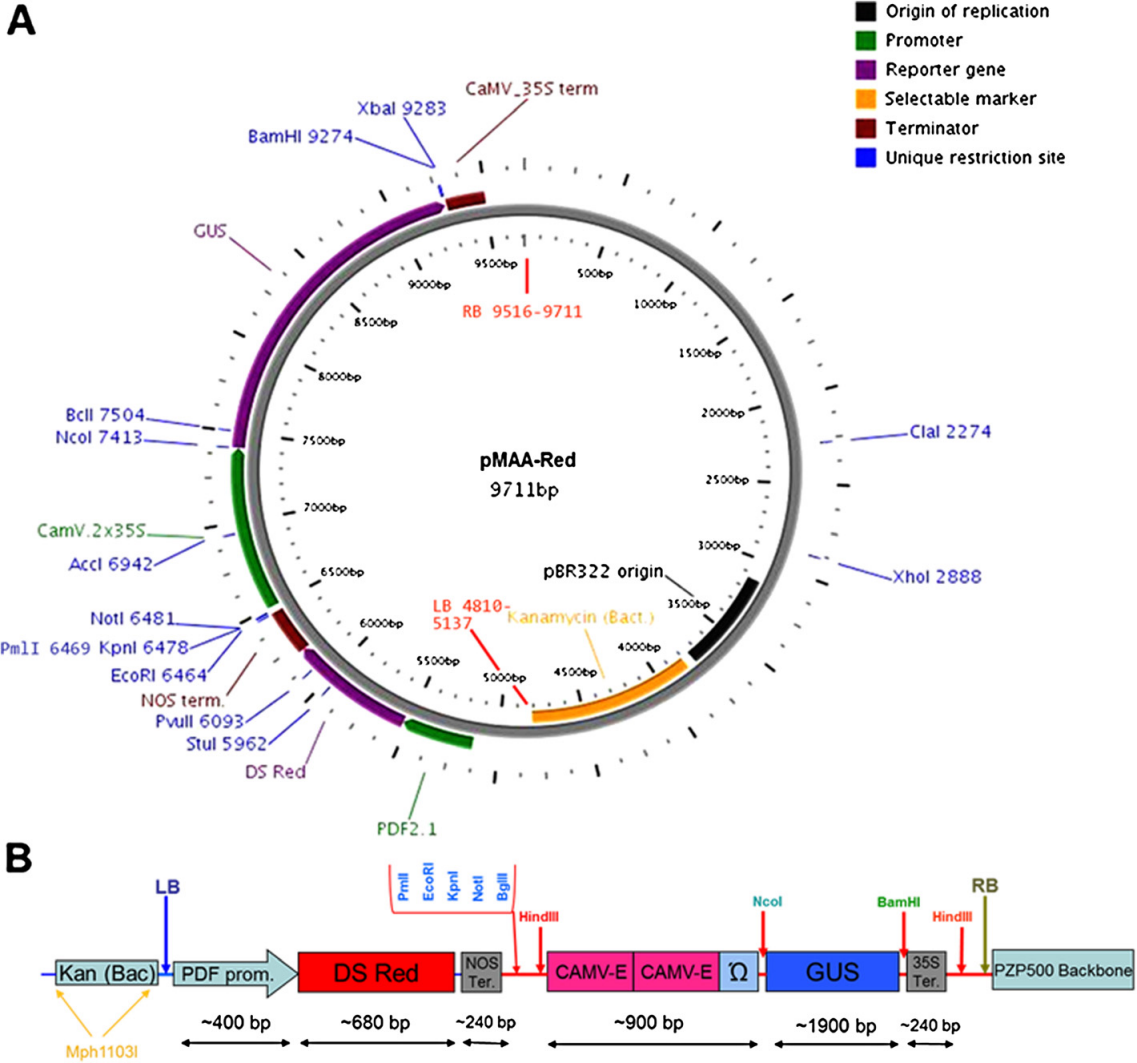
**pMAA-Red. **Circular map with main restriction sites and polylinker (**A**), (**B**) Diagram showing the length of different fragments and restriction sites.

Vectors containing a fluorescent protein coupled to a seed-specific promoter have been described before. Here, we have used the fluorescent marker DsRed [14,22] and the promoter of the *Pdf2.1* gene [20] from Arabidopsis because we were especially interested in overexpressing genes that are downregulated in syncytia induced by the beet cyst nematode *H. schachtii* in Arabidopsis roots [23]. The reason to use this promoter was that it is not only expressed in seeds but also in syncytia [21]. The *Pdf2.1* promoter (~400 bps) and DsRed region were amplified from the vector pPZP3425-pPDF2.1:: DsRed (Bohlmann, unpublished) which contained a *Pdf2.1* promoter fused to DsRed. The *Pdf2.1* promoter was amplified using the pPDF2.1EcoFor primer containing an EcoRI site and the pPDF2.1NcoRev primer with NcoI site (Table 1). For amplification of the DsRed + 35 S terminator fragment, Redfor with BspHI and Redrev with BamHI sites were used. The *PDF2.1* promoter PCR product was digested with EcoRI and NcoI and that of DsRed with BspHI and BamHI. The vector pPZP600 was digested with EcoRI and BamHI. These three fragments were mixed and ligated together to yield pPZP650 (Additional file 2: Figure S2). The EcoRI and BamHI sites were eliminated sequentially by digestion, filling in the ends using Klenow enzyme, and religation which resulted in the intermediate vector pPZP650.3 (not shown). This intermediate vector was digested with HindIII and a polylinker (PmlI, EcoRI, KpnI, NotI, BglII and HindIII sites provided by oligos Linker1 and 2) was inserted. The orientation of the polylinker was determined by digestion with EcoRI and XbaI (only present in the terminator downstream of the HindIII site). This vector was named pPZP653 (Additional file 2: Figure S2). We then introduced the GUS cassette from PZP3425 by digesting pPZP653 with HindIII and ligating the vector with the GUS cassette derived also by digestion with HindIII to make pPZP6535 (Additional file 2: Figure S2). Orientation of the cassette was tested by PCR using DsRedfor2 and GUSrev primers (Table 1). Since this vector contained two 35 S terminators we could not use terminator reverse primers for sequencing of our gene of interest delivered by the CaMV promoter. We therefore, replaced the 35 S terminator in the DsRed cassette with a *nos* terminator by a series of polymerase chain reactions. In a first step, the last part of DsRed (containing a StuI site) from vector pPZP6535 was amplified by using primers DsRedMfor and DsREDnosTERrev. In the second step, we amplified the *nos* terminator by using primers DsREDnosTERfor primer and nosTERrevEco (with EcoRI site). Then overlapping PCR was done using DsRedMfor and nosTERrevEco primers. Both, the amplified PCR product and pPZP6535 were digested with StuI and EcoRI and large vector fragment and insert were ligated to produce the final binary vector pMAA-Red (MAA is named after the initials of first author name and Red for Ds-Red) (Figure 3A and B). 

**Table 1:** List of oligonucleotides used in this work

**No**	**Name**	**Sequence**
1	PZPN-1/Kpn2Ifor	GGCTCCGGAGAATGAACGCCAA
2	PZPN-1rev	TTCGATCAGCGGTTGCTTGCCA
3	PZPN-2for	TCGTGGCAAGCAACCGCTGAT
4	PZPN-2/XhoIrev	GCCTCGAGAGGCCGACGCGA
5	PZPN-1/Kpn2Ifor	GGCTCCGGAGAATGAACGCCAA
6	PZPN-2/XhoIrev	GCCTCGAGAGGCCGACGCGA
7	PZP2/XhoIfor	CAGACTCGAGTGTACTGAGAGTGCAC
8	PZP2/Kpn2Irev	AACGTCCGGAGCCGACTGCACTATAGCA
9	P500Kp1Nseqfor	ATTCGAGCTCGGTACCCGGG
10	P500N2seqfor	TTAGCGGCTAAAGGAGGCGG
11	P500Xh3Nseqfor	AGGTCTCTTTCCTGTGGATAGC
12	P500for	GCCCGAGGCATAGACTGTAC
13	P500rev	GCATCAGACAAACCGGCCAG
14	PZP500Mphfor	GAATGCATCACAGGCAGCAACGCT
15	PZP500Mphrev	GCGTGCATAATAAGCCCTACA
16	KanforMph	CAGCATCATGCATAATTGTGGTTTCA
17	KanrevMph	GTTGCGATGCATCTAGGTACTAAAACAAT
18	pPDF2.1EcoFor	AATGAATTCCAGAATGAGTTGTCA
19	pPDF2.1NcoRev	CATAGAGAACTCCATGGTTGGAGAAAG
20	RedforBspHI	AGATCATGACCTCCTCCGAGAAC
21	RedrevBam	AGTCGGATCCGCTAGAGGAACAGGT
22	DsRedfor2	CCACCACCTGTTCCTCTAGC
23	Linker1	AGCTGAATTCACGTGGTACCGCGGCCGCAGATCTA
24	Linker2	AGCTTAGATCTGCGGCCGCGGTACCACGTGAATTC
25	P500terfor	GCTGGTGGCAGGATATATTG
26	P500RBseqrev	TTAAACTGAAGGCGGGAAAC
27	DsRedMfor	CAGAAGAAGGACAATGGGCTGGGA
28	DsREDnosTERfor	ACCTGTTCCTCTAGGATCGTTTCAAAC
29	DsREDnosTERrev	TTTGAACGATCCTAGAGGAACAGGTG
30	nosTERrevEco	TGAGAATTCAGAGATCTAGTAACATAG

### Production of transgenic Arabidopsis lines using pMAA-Red

**Figure 4:**
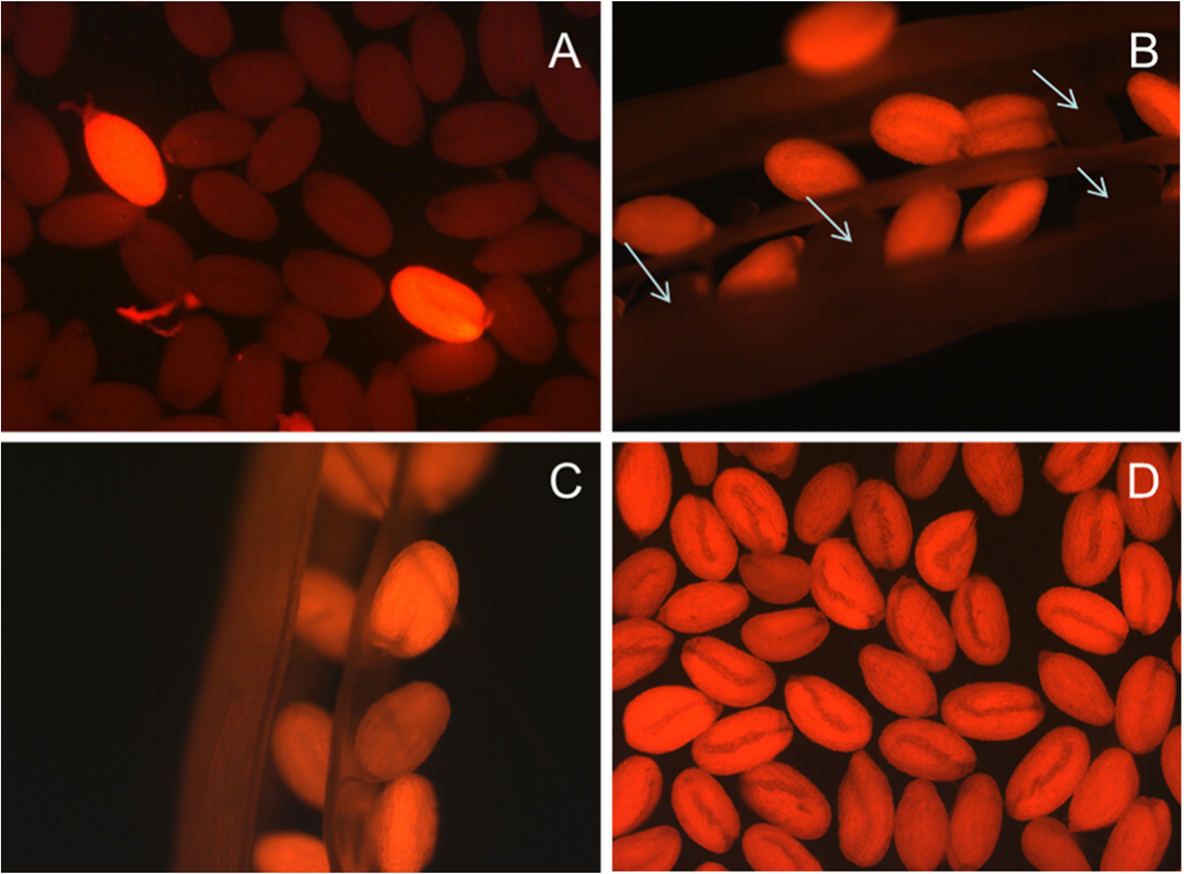
**pMAA-Red seed selection. **Fluorescent seeds from transgenic Arabidopsis lines transformed with pMAA-Red (**A**) Selection of transformed seed (T_0_) under microscope, (**B**) Silique in T_1_ generation shows some non-florescent seeds (arrows) representing heterozygous line, (**C**) Silique in T_2_ generation shows all florescent seeds representing homozygous line, (**D**) The selected homozygous line in T_2_ generation.

**Figure 5:**
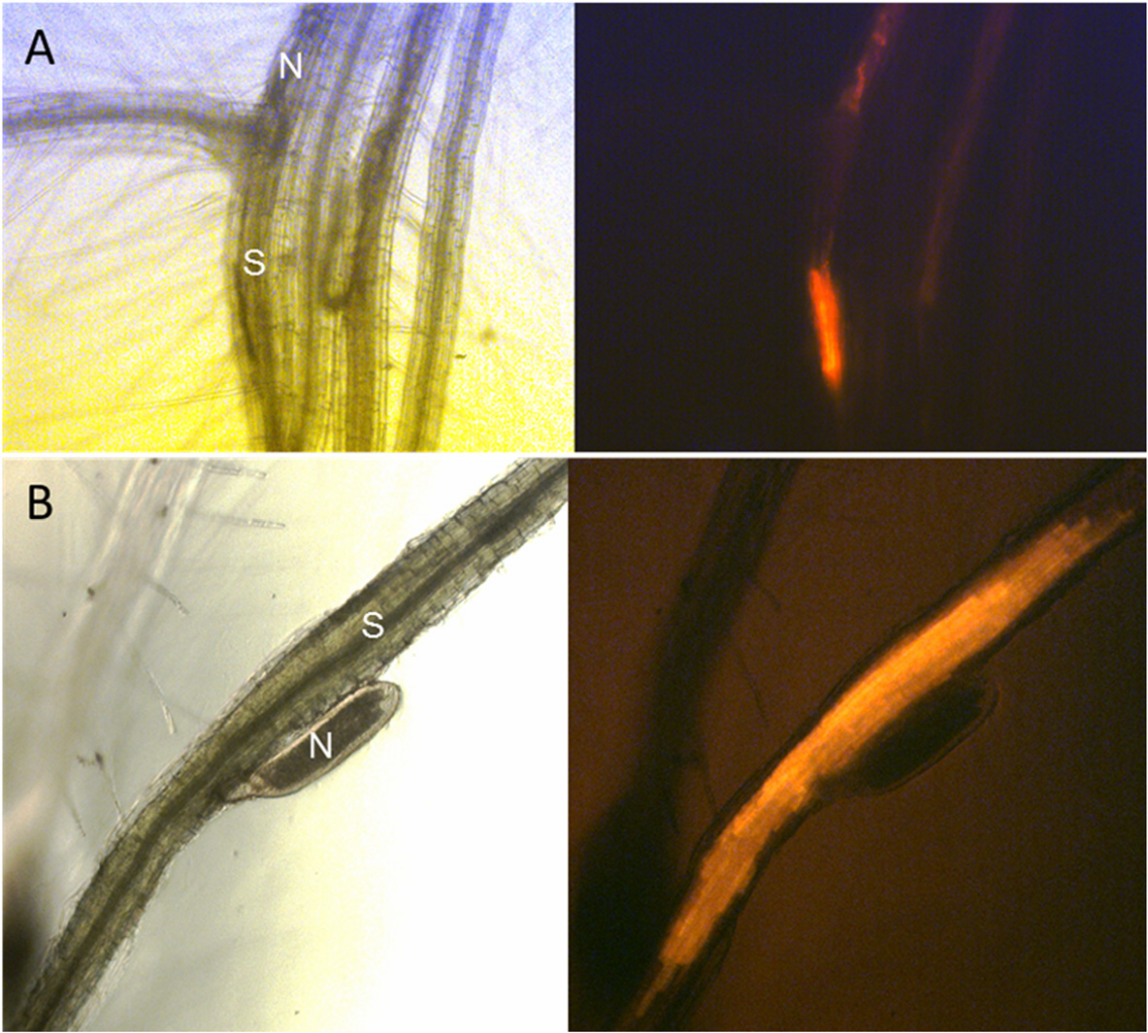
**DsRed fluorescence in syncytia. **Syncytia induced by *H. schachtii *in roots of an Arabidopsis line transformed with pMAA-Red show DsRed florescence at 5 dpi (**A**) and 10 dpi (**B**). Left, bright field image; right DsRed fluorescence.

The vector pMAA-Red was transformed into Agrobacteria and then into Arabidopsis using the floral dip method. Seeds were harvested and red fluorescent seeds were selected using an inverted fluorescence microscope (Figure 4A). These T1 seeds were grown on soil and siliques that showed a 3:1 ratio of fluorescent and non-fluorescent seeds (Figure 4B) were used to produce homozygous lines (Figure 4C and D) which did not show any phenotypic difference. We confirmed the expression of the DsRed cassette in syncytia by growing seedlings from homozygous lines on agar and infection with *H. schachtii* larvae. As expected, in these lines, the *Pdf2.1* promoter directed DsRed expression not only in seeds but also in syncytia (Figure 5A and B).

**Figure 6:**
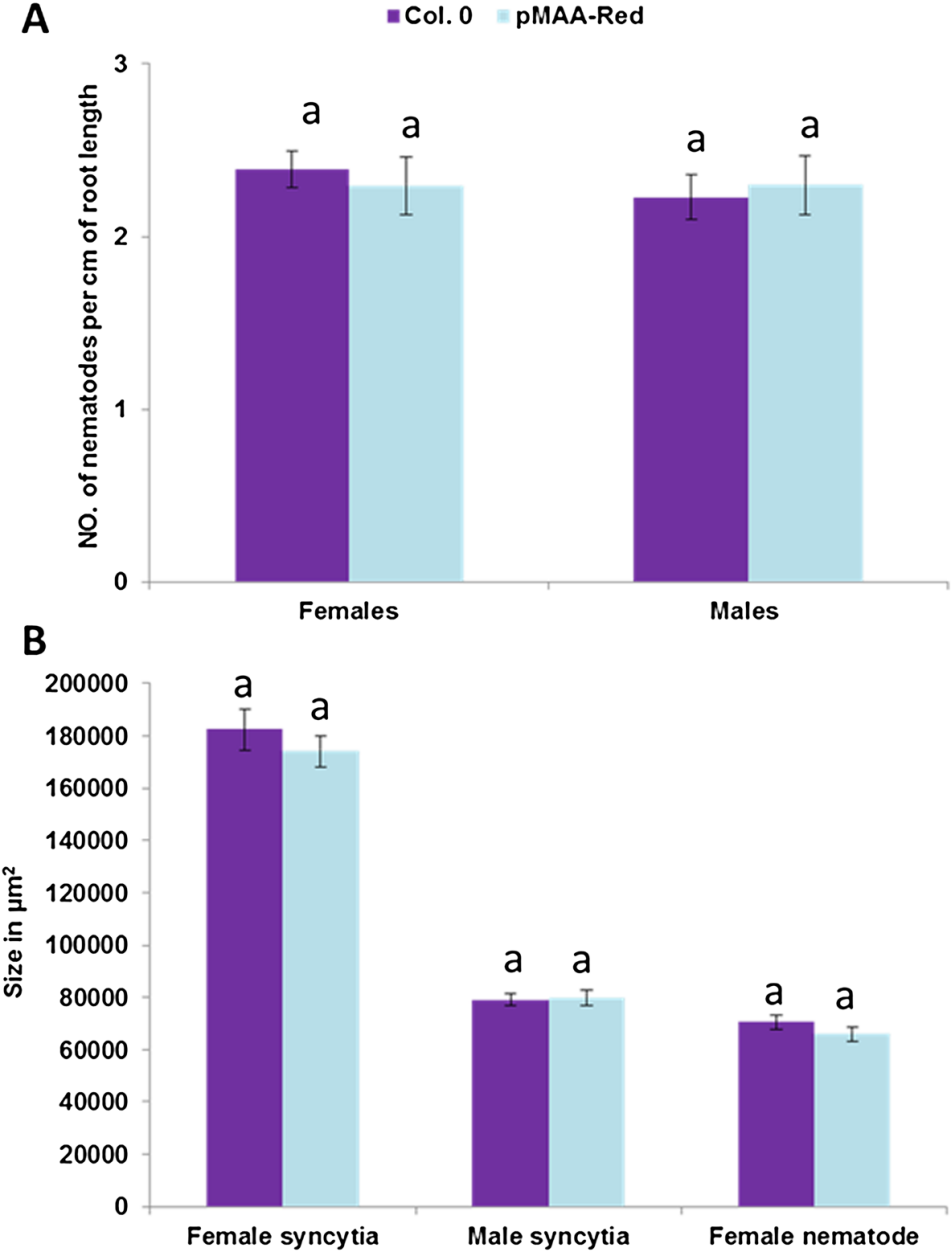
**Nematode infection test. **Infection assay of a homozygous line transformed with pMAA-Red compared with wild-type (Col.0) plants. (**A**) Number of male and female nematodes per cm of root was determined at 15 dpi showing no significant differences between wild-type and the vector line (P < 0.05, one way ANOVA and LSD). The statistical significance was determined from three independent experiments each having 5 plates with 10 plants per plate. Values are means ± SE, n = 15. (**B**) Size of female syncytia, male syncytia, and female nematodes at 15 dpi was not different between the vector line and the wild-type (P < 0.05, one way ANOVA and LSD). Values are means ± SE, n = 30.

It could be argued that the DsRed expression in syncytia might influence the development of syncytia and lead to higher susceptibility or resistance. To test this possibility, we compared the resistance of an Arabidopsis line transformed with the vector pMAA-Red with wild-type plants at 15 dpi. We could not detect significant differences between wild-type plants and the transgenic line as numbers of males and females as well as sizes of syncytia and female nematodes are concerned (Figure 6A and B).

**Figure 7:**
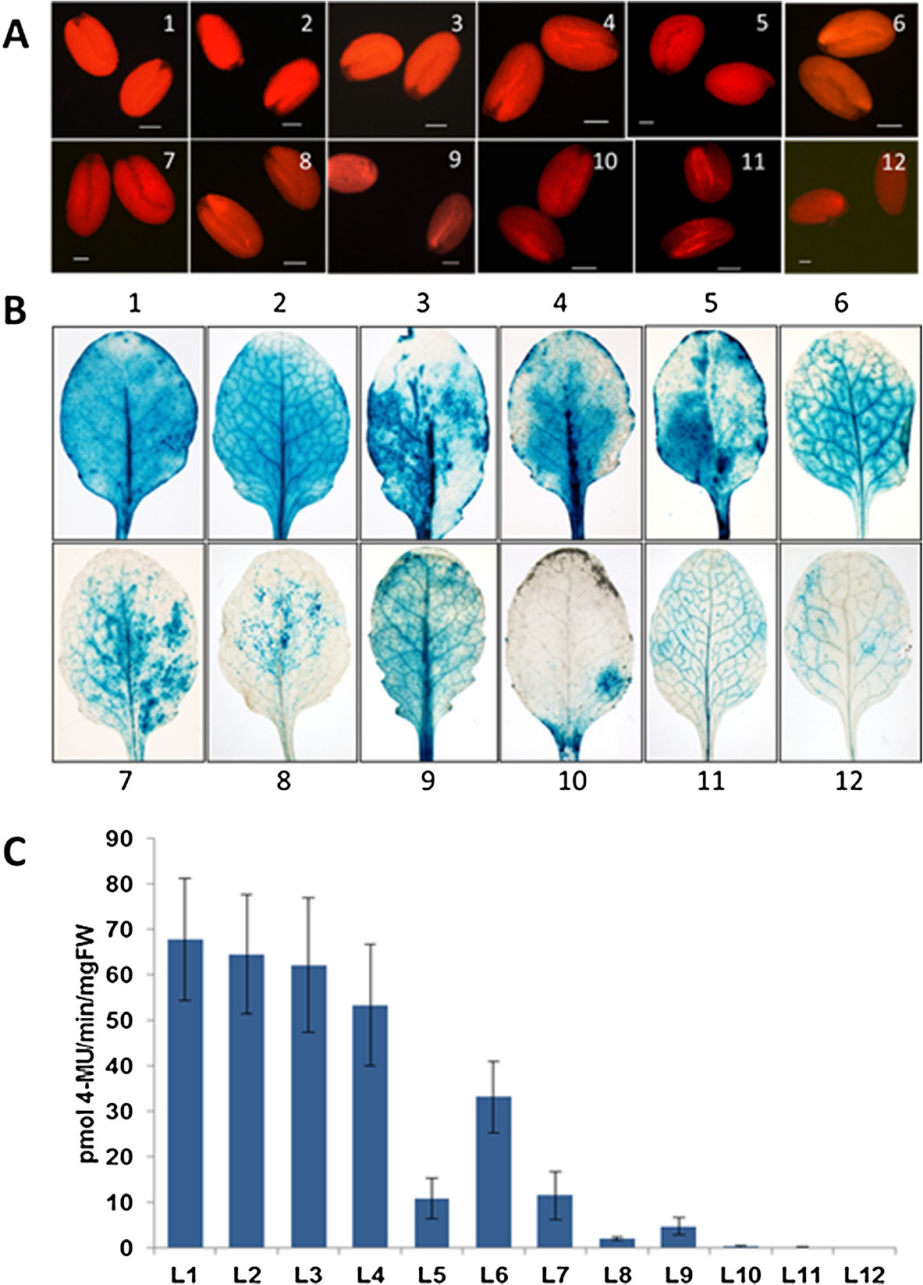
**Selection of lines with a strong expression level. **Seeds from 12 transgenic lines of pMAA-Red were arranged according to decreasing fluorescence (**A**). Histochemical GUS staining of leaves from these lines is shown in (**B**) and GUS quantification of seedlings in (**C**).

Overexpression lines are often produced to achieve a strong expression of the gene of interest. For that reason strong promoters are used, such as the CaMV 35 S promoter [24]. However, mainly due to the insertion of the transgene in different parts of the genome, the transgenic lines that are produced vary widely in expression level [24]. It is therefore necessary to screen a number of lines at the transcript level or the protein level. We reasoned that this lengthy procedure could be shortened if selecting those lines with a strong fluorescence in seeds. To test this hypothesis, we selected 12 transgenic lines transformed with the vector pMAA-Red. We arranged these lines by eye according to the strength of fluorescence in the seeds (Figure 7A). We grew seedlings of these lines and stained the leaves for GUS expression (Figure 7B) which showed a correlation with DsRed expression in seeds. We confirmed this result by measuring the GUS expression level in seedlings (Figure 7C). Thus, to select transgenic lines with a strong expression level, one simply has to select several transgenic seeds with a strong DsRed fluorescence. 

## Discussion

A large variety of binary vectors for plant transformation have been described. Among them the pPZP series of vectors [6] and the derived pCAMBIA vectors [7] are especially popular. A prominent feature of these vectors is their stability in bacteria, the high copy number, and the relatively small size. One disadvantage was the use of the CaMV promoter for the plant selectable marker and the use of spectinomycin and chloramphenicol as selectable markers for bacteria which led us to construct the vector pPZP3425 [8]. In this vector the CaMV35S promoter for the plant selectable marker (kanamycin) was replaced by the weaker *nos* promoter and a kanamycin resistance gene for selection in bacteria was included in the vector backbone.

Although pPZP3425 proved useful for our purposes in producing promoter:: *GUS* lines (by replacing the CaMV promoter in the *GUS* cassette with a promoter of interest) or overexpression lines (by replacing *GUS* in the *GUS* cassette with a gene of interest) [8], selection of homozygous transgenic lines with a strong expression of the gene of interest was still a lengthy procedure. Since we were interested to produce a large number of transgenic Arabidopsis overexpression lines with a strong expression level for putative antimicrobial peptides we set out to construct a pPZP vector that would have three important features: First, it should allow us to use selection or screening on soil to avoid growing Arabidopsis under sterile conditions. Second, we wanted to easily select lines with a strong expression level. Third, the vector should be used for the expression in syncytia using specific promoters that would not be active in seedlings or leaves.

The first precondition excluded the use of antibiotic resistance such as kanamycin or hygromycin or the use of metabolite resistance genes such as the *E. coli-*derived phosphomannose isomerase which allows growth on mannose [25] or the *Streptomyces rubiginosus* xylose isomerase (*xylA*) gene which allows growth on xylose [26] as a plant selectable marker for which sterile conditions would have to be used. Herbicide resistance would avoid the need for sterile growth but all T1 seedlings have to be grown for selection. In contrast, if using a fluorescent marker that is expressed in seeds, only the transgenic seeds obtained after transformation would have to be grown further and no additional treatment would be needed. Such an approach is especially important if a large number of transgenic lines have to be produced. An example is the work of [27] who conducted a high-throughput screen in Arabidopsis for castor genes that would lead to changes in hydroxy fatty acid composition in seeds. We decided to try the DsRed gene as a screenable marker [13-17]. This leaves the possibility to use in addition GFP or other most frequently used fluorescent markers as reporter genes. Furthermore, DsRed has a rather weak fluorescence which might seem to be a disadvantage. However, this weak fluorescence makes it easier to identify seeds with a different level of fluorescence.

Expression of the gene of interest varies largely in independent transgenic lines [24] due mainly to position effects. One way of reducing this variation is the inclusion of matrix attachment regions in the T-DNA. However, this only worked with gene silencing mutants, which limits the use of these vectors [28]. We have demonstrated here that the use of the vector pMAA-Red allows an easy and efficient selection of transgenic lines with a strong expression of the gene of interest in wild-type Arabidopsis plants. Of course, mutants could also be used, if needed. Pre-selection of lines with a strong DsRed expression in seeds according to their fluorescence reduces the number of lines that have to be tested at the transcript or protein level.

Our third precondition was that the vector should allow the use of syncytium-specific promoters instead of the CaMV promoter which is active in most tissues of Arabidopsis plants. We have recently shown that several genes are expressed in syncytia which are normally expressed in pollen [23], such as *MIOX4* and *MIOX5*, or in seeds, such as *Pdf2.1* whose promoter is used here to drive the expression of DsRed in seeds. Using the *MIOX4* or *MIOX5* promoter would allow a specific expression in syncytia which could be useful for genes with a negative effect on plant growth. However, screening such transgenic lines would require the analysis of expression of the gene of interest in syncytia. Such a screening is very time consuming because syncytia have to be cut out from infected roots. In this case a pre-screening that would reduce the number of lines would lead to a significant reduction of time and effort. Thus, the pMAA-Red vector that we have constructed is especially useful for the syncytium-specific expression of transgenes or for similar cases where the expression of the transgene would be restricted to tissues that could not be easily screened. Promoter::reporter constructs and overexpression lines can be produced from this vector as from pPZP3425 [8]. The CaMV promoter can be replaced by a promoter of interest using NcoI and one of the unique sites in the polylinker. The GUS sequence can be replaced with a sequence for overexpression of a gene of interest by using NcoI and BamHI. If the sequence to be cloned contains NcoI or BamHI site, it is usually possible to use a restriction enzyme that produces compatible cohesive ends with NcoI and BamHI. For instance, BspHI, PciI, and FatI produce cohesive ends that are compatible with NcoI.

After transformation of Arabidopsis plants it takes about 3–4 weeks until T1 seeds can be harvested and inspected for fluorescent seeds. A big advantage of transformation with the vector pMAA-Red is that only these selected seeds have to be grown on soil to produce the T2 generation. After another 4 weeks, the first siliques of these plants can be screened for a 3:1 segregation of fluorescent seeds which can then be used to produce homozygous T3 seeds for further analysis. Again, the first siliques of these plants can be used to select homozygous lines and only those will be grown for maturity, which will take a total of 6 to 8 weeks. Depending on the growth conditions, the whole procedure from transformation to harvesting homozygous seeds could be completed within four month.

## Conclusion

We have constructed compact pPZP vectors without NotI sites having either bacterial spectinomycin or kanamycin resistance (pPZP500 and pPZP600, respectively) and a vector (pMAA-Red) which allows an easy production of transgenic Arabidopsis overexpression lines with strong expression levels of the gene of interest.

## Methods

### Cloning methods

Standard procedures were used for restriction enzyme mediated DNA digestion, ligation, and transformation [29]. Restriction enzymes and T4 DNA ligase were from Fermentas -Thermo Fisher Scientific and New England Biolabs. The *E coli* strain DH10B was used throughout this work. PCR was done using Eppendorf Mastercycler Gradient.

### Construction of pPZP500

The NotI sites in pPZP200 were eliminated by a series of PCR amplifications using pPZP200 as template. Ligation of the amplified fragments yielded a binary vector without NotI sites. The outline of this construction and the position of primers is given in Additional file 1: Figure S1. In a first round of PCR reactions, a 1549 bp backbone fragment from pPZP200 was amplified using primers PZPN-1/Kpn2Ifor and PZPN-1rev (see Table 1 for primers and Table 2 for vectors used in this work) and a 1283 bp fragment was amplified by using primers PZPN-2for and PZPN-2/XhoIrev. Both fragments were mixed and used as template in a second PCR to amplify a 2824 bp fragment (first half of pPZP200 i.e., so called insert) by using primers PZPN-1/Kpn2Ifor and PZPN-2/XhoIrev. In another PCR reaction the remaining half (2814 bp) of pPZP200 (i.e., so called vector) was amplified by using primers PZP2/XhoIfor and PZP2/Kpn2Irev. Vector and insert fragment were digested with Kpn2I and XhoI and ligated to yield pPZP500 (Figure 1) that was transformed in *E. coli* for selection purpose and the modified parts were sequenced using primers P500Kp1Nseqfor, P500N2seqfor, P500Xh3Nseqfor, P500for, and P500rev. To further confirm that after removing some sites from the backbone of pPZP200 the new pPZP500 vector is functional, we have cloned a GUS cassette (containing a duplicated CaMV 35 S promoter with omega element, *uidA* gene with intron, and CaMV terminator) from pPZP3425 [8] as HindIII fragment into the same site of pPZP500 to produce pPZP5025 (Additional file 2: Figure S2). This plasmid was transformed into Agrobacteria which were used for agroinfiltration into leaves of *N. benthamiana.* Four days after infiltration, leaves were processed for GUS staining, indicating full functionality of the vector (Figure 2). 

**Table 2:** Vectors used in this study

**No**	**Name**	**Features**	**Reference**
1	pPZP200 (Additional file 1: Figure S1)	Spectinomycin resistance gene for bacterial slection	[6]
2	pPZP3425	CaMV 35 S 2X promoter with omega element, LacZ operon, GUS, CaMV terminator (GUS cassette), kanamycin resistance gene for bacterial and plant selection	[8]
3	pPZP500 (Figure 1)	pPZP200 modified backbone without NotI sites	This study
4	pPZP5025 (Additional file 2: Figure S2)	pPZP500 with GUS cassette	This study
5	pPZP3425-pPDF2.1::DsRed	pPZP3425 containing pPDF2.1::DsRed instead of the 35 S::GUS cassette	Bohlmann, unpublished
6	pPZP600 (Additional file 2: Figure S2)	pPZP500 with kanamycin resistance gene for bacterial selection	This study
7	pPZP650 (Additional file 2: Figure S2)	pPZP600 with *PDF2.1 *promoter, DsRed gene, and 35 S terminator	This study
8	pPZP653 (Additional file 2: Figure S2)	pPZP650 with new polylinker	This study
9	pPZP6535 (Additional file 2: Figure S2)	pPZP653 with GUS cassette	This study
10	pMAA-Red (Figure 3)	pPZP6535 with *PDF2.1 *promoter, DsRed gene, *nos *terminator and GUS cassette	This study

### Construction of pPZP600

The spectinomycin resistance gene of pPZP500 was replaced by the *nptII* gene. The *nptII* gene (~1 kb) was amplified from pPZP3425 using KanforMph and KanrevMph primers. The pPZP500 vector backbone was amplified by using PZP500Mphfor and PZP500Mphrev primers. Both primers had a Mph1103I site at their ends. Insert and vector fragment were digested with Mph1103I*,* ligated, and transformed into *E. coli* to yield pPZP600 (Additional file 2: Figure S2).

### Plant growth

*N. benthamiana* and Arabidopsis plants were grown in a growth chamber at 25 ± 1°C, with a 16 hour light/8 hour dark photoperiod and approximately 65% humidity.

### Agrobacterium transformation

Binary plasmids were transformed into *A. tumifaciens* strain GV3101 by the freeze-thaw method [30]. Agrobacteria were selected on YEB plates with appropriate antibiotics which included 25 μg/ml gentamycin and 35 μg/ml rifampicin for Agrobacteria. Selection for binary plasmids was done with 200 μg/ml spectinomycin (for pPZP5025) or 50 μg/ml kanamycin (for pMAA-Red).

### Agroinfiltration of N. Benthamiana

Agrobacteria were grown to an OD_600_ of 0.8 overnight in an incubator/shaker at 28°C. Bacteria were harvested by centrifugation at 5000 rpm for 6 min in a table top centrifuge at room temperature and suspended in infiltration medium (10 mM MES pH 5.6, 10 mM MgCl_2_ and 100 μM acetosyringone) to obtain bacterial suspensions of the an OD_600_ of 1.0. After incubation for 2 hr at room temperature, Agrobacterium suspensions were infiltrated in the abaxial side of leaves by using a 1 ml syringe without a needle. Infiltrated plants were kept under the same growth conditions as mentioned above. For co-infiltration of the RNA silencing inhibitor P19, an equal volume of a bacterial suspension harbouring pBin61P19 [31] was added prior to infiltration.

### Arabidopsis transformation

The vector construct pMAA-Red was transformed into Arabidopsis ecotype Col by the floral dip method [32]. Seeds of transformed plants were harvested and photographed under an inverse microscope equipped with a DsRed fluorescence filter (Axiovert 200 M; Zeiss, Hallerbergmoos, Germany) and an integrated camera (AxioCam MRc5; Zeiss). Fluorescent seeds (T_1_) were selected and put on soil to grow the next generation (T_2_), of which the first mature silique was detached from the plant and examined under the microscope for fluorescent seeds to check for 3:1 segregation. These seeds were then used to produce homozygous lines. Seeds that were kept in the dark at 4°C still showed fluorescence after several years.

### GUS staining

GUS staining of the *N. benthamiana* leaves was done by overnight incubation in X-Gluc solution (50 mM sodium phosphate buffer pH 7.0, 10 mM EDTA pH 8.0, 0.1% (v/v) Triton X-100 and 0.5 mg/ml X-Gluc) at 37°C [33] followed by several washings with 70% ethanol to remove the chlorophyll from leaf tissues.

### GUS activity assay

GUS activity was measured according to Jefferson et al. [33] with some modifications in black 96-well Greiner plates. To 100 μl of total protein extract 50 μl of 4 mM 4-MUG was added. The reaction was incubated at 37°C for 5 min and then 50 μl of 0.5 M Na_2_CO_3_ was added to stop the reaction. Fluorescence was measured at 355 nm excitation and 460 nm emission in a FLUOstar Omega micro plate reader (BMG Labtech) using 4-MU standards (10 mM stock in ethanol and diluted in GUS extraction buffer) in the range of 1–100 μM.

### Nematode infection and examination of fluorescence in syncytia

The seeds of a pMAA-Red transgenic Arabidopsis line were surface-sterilized for 7 min in 10% Chlorox (v/v), submerged for 5 min in 70% (v/v) ethanol, and then washed three times in sterile water. The sterilized seeds were placed on a modified 0.2 concentrated Knop agar medium supplemented with 2% sucrose [34]. *H. schachtii* was multiplied *in vitro* on mustard (*Sinapsis alba* cv. Albatros) roots growing on 0.2 concentrated Knop medium supplemented with 2% sucrose [34]. Hatching of J_2_ larvae was stimulated by soaking the cysts in sterile 3 mM ZnCl_2_. The juveniles were washed four times in sterile water and resuspended in 0.5% (w/v) Gelrite for inoculation. Roots of 12-day-old Arabidopsis plants were inoculated with about 40–50 juveniles under sterile conditions. At 5 and 10 dpi the fluorescent syncytia were examined using an inverse microscope.

### Resistance tests and size measurement of syncytia and nematodes

*H. schachtii* (Schmidt) infection was done in the same way as described above. Roots of 12-day old Arabidopsis plants were inoculated under axenic conditions with about 50 juveniles per plant. The number of male and female nematodes per cm of root length were counted at 14 dpi. The data were analysed using single factor ANOVA (P < 0.05). As the F-statistic was greater than F-critical, a Least Significance Test (LSD) was applied. At 14 dpi, pictures of male syncytia, female syncytia and females nematodes (30 photos for each) were taken. Syncytia and nematodes were measured using an inverse microscope (Axiovert 200 M; Zeiss, Hallerbergmoos, Germany). The data were analyzed using single factor ANOVA (P < 0.05).

## Competing interests

The authors declare that they have no competing interests.

## Authors' contributions

MAA cloned pMAA-Red and did the nematode experiments. KHS cloned pPZP500 and did the GUS assays. MAA and KHS helped to draft the manuscript. HB conceived and coordinated the study and helped in writing the manuscript. All authors read and approved the final manuscript.

## GenBank accession numbers

pPZP500: JX025644

pPZP600: JX025643

pMAA-Red: JX025642

## Supplementary Material

Additional file 1 **Figure S1. **Position of Primers for*NotI *Elimination in pPZP200. Click here for file

Additional file 2 **Figure S2. **The various intermediate constructs made during the construction of pMAA-Red from pPZP600. Click here for file
