# Supplementary figures and images for: pMAA-Red: a new pPZP-derived vector for fast visual screening of transgenic Arabidopsis plants at the seed stage
Source: BMC Biotechnol. 2012 Jul 2;12:37. doi: 10.1186/1472-6750-12-37 (PMC3478159; doi:10.1186/1472-6750-12-37)

Figure S1: Position of Primers for *NotI* Elimination in pPZP200


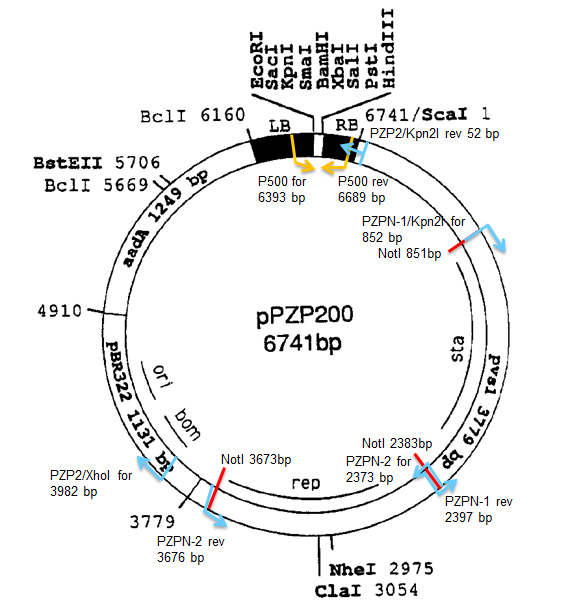

Supplement: Additional file 1 — Figure S1. Position of Primers forNotI Elimination in pPZP200. [file 1472-6750-12-37-S1.docx]

Figure S2: The various intermediate constructs made during the construction of pPZP6635 from pPZP600


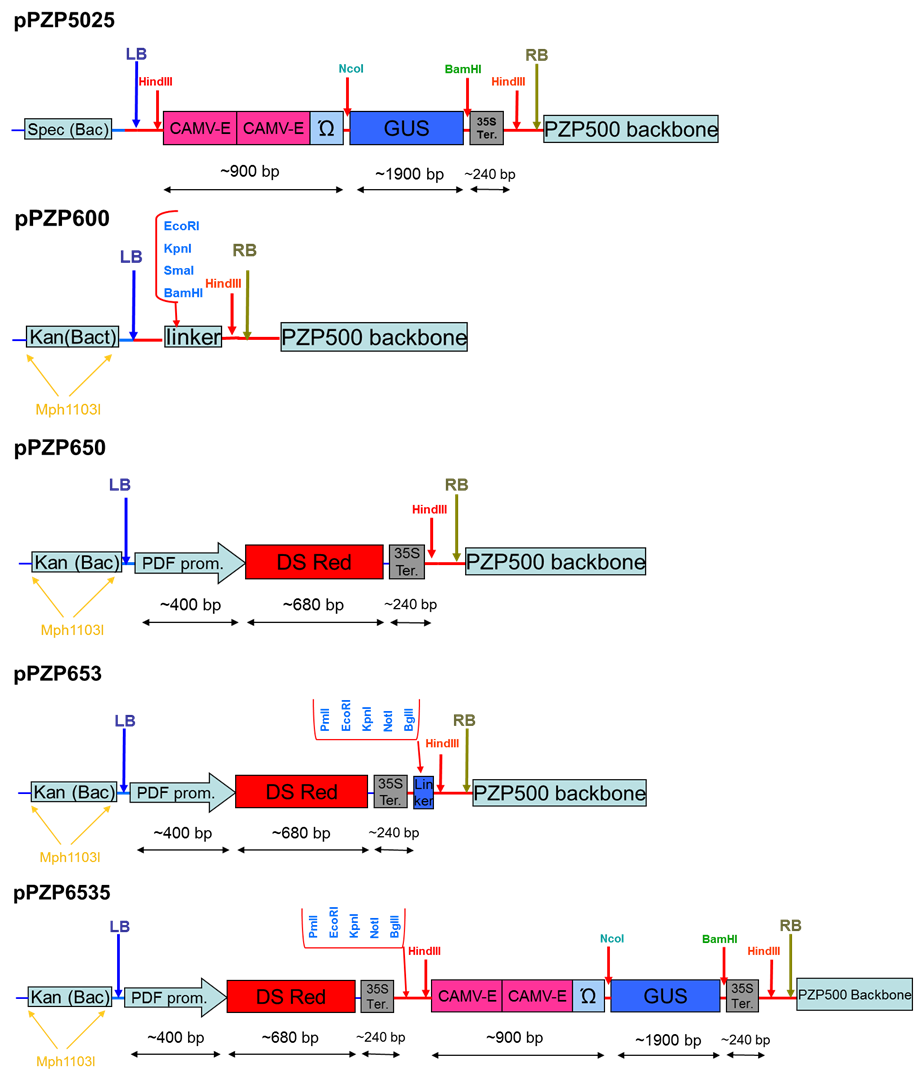

Supplement: Additional file 2 — Figure S2. The various intermediate constructs made during the construction of pMAA-Red from pPZP600. [file 1472-6750-12-37-S2.docx]
